# Small Pox in Washington

**Published:** 1863-12

**Authors:** 


### SMALL POX IN WASHINGTON.

(Extract from a private letter.)

This city is full of small Pox. I presume you have seen in the papers that the President has had varioloid. It is not wonderful that it should invade high places, when such reckless neglect is practiced as occurs in this city. No precautions are used. If any person gets disease he is kept at home, and no card of warning put up, or precaution used, to prevent its spread.

I may overstate, but it has been said to me that there are 400 cases of this loathsome disease now in the city. The contrabands are a fruitful source of propagation; being poor, they invariably wear their old clothes after recovery, and thus continue to spread the disease.

